# Combination of R-R Interval and Crest Time in Assessing Complexity Using Multiscale Cross-Approximate Entropy in Normal and Diabetic Subjects

**DOI:** 10.3390/e20070497

**Published:** 2018-06-27

**Authors:** Ming-Xia Xiao, Hai-Cheng Wei, Ya-Jie Xu, Hsien-Tsai Wu, Cheuk-Kwan Sun

**Affiliations:** 1 School of Electrical and Information Engineering, North Minzu University, No. 204 North—Wenchang St., Xixia District, Yinchuan 750021, China; 2 School of Computer and Information, Hefei University of Technology, No. 193, Tunxi Rd., Hefei 230009, China; 3 Department of Electrical Engineering, National Dong Hwa University, No. 1, Sec. 2, Da Hsueh Rd., Shoufeng, Hualien 97401, Taiwan; 4 Department of Emergency Medicine, E-Da Hospital, I-Shou University School of Medicine for International Students, No.1, Yida Road, Jiaosu Village, Yanchao District, Kaohsiung City 82445, Taiwan

**Keywords:** multiscale entropy (MSE), cross-approximate entropy, crest time, R-R interval, diabetes

## Abstract

The present study aimed at testing the hypothesis that application of multiscale cross-approximate entropy (MCAE) analysis in the study of nonlinear coupling behavior of two synchronized time series of different natures [i.e., R-R interval (RRI) and crest time (CT, the time interval from foot to peakof a pulse wave)] could yield information on complexity related to diabetes-associated vascular changes. Signals of a single waveform parameter (i.e., CT) from photoplethysmography and RRI from electrocardiogram were simultaneously acquired within a period of one thousand cardiac cycles for the computation of different multiscale entropy indices from healthy young adults (n = 22) (Group 1), upper-middle aged non-diabetic subjects (n = 34) (Group 2) and diabetic patients (n = 34) (Group 3). The demographic (i.e., age), anthropometric (i.e., body height, body weight, waist circumference, body-mass index), hemodynamic (i.e., systolic and diastolic blood pressures), and serum biochemical (i.e., high- and low-density lipoprotein cholesterol, total cholesterol, and triglyceride) parameters were compared with different multiscale entropy indices including small- and large-scale multiscale entropy indices for CT and RRI [MEI_SS_(CT), MEI_LS_(CT), MEI_SS_(RRI), MEI_LS_(RRI), respectively] as well as small- and large-scale multiscale cross-approximate entropy indices [MCEI_SS_, MCEI_LS_, respectively]. The results demonstrated that both MEI_LS_(RRI) and MCEI_LS_ significantly differentiated between Group 2 and Group 3 (all *p* < 0.017). Multivariate linear regression analysis showed significant associations of MEI_LS_(RRI) and MCEI_LS_(RRI,CT) with age and glycated hemoglobin level (all *p* < 0.017). The findings highlight the successful application of a novel multiscale cross-approximate entropy index in non-invasively identifying diabetes-associated subtle changes in vascular functional integrity, which is of clinical importance in preventive medicine.

## 1. Introduction

The World Health Organization has identified cardiovascular and cerebrovascular diseases as the top two global killers. The development of such “non-communicable diseases” is attributable to chronic metabolic anomalies, including hyperglycemia, hyperlipidemia, and hypertension, that require early detection and timely intervention [1]. In addition to commonly available non-invasive monitoring parameters such as heart rate, blood pressure, and electrocardiogram (ECG), more sophisticated and accurate indicators of changes in microvascular blood flow are needed for guiding lifestyle modifications before the development of overt diseases [2]. Aged and hypertensive subjects were first reported to show carotid arterial pulsations different from those of healthy individuals over five decades ago [3]. A decade later, analog and electrical arterial pulsation signals were found to be consistent, thereby validating non-invasive means of arterial stiffness analysis using waveform contour [4]. A large-scaled study later demonstrated age-related differences in arterial waveform contour [5]. With the advancement of electronic technology, photoplethysmography (PPG) has become a popular means of acquiring “digital volume pulse” (DVP) that accurately reflects peripheral microvascular blood flow. Changes in DVP have been reported under physiological [6] and pathological [7] conditions.

Apulse wave is a complex physiological signal composed of systolic and diastolic components. The former arises mainly from aforward-going pressure wave transmitted from the left ventricle to the recording site (i.e., finger), while the latter arises mainly from pressure waves transmitted along the aorta to small arteries in the lower body, from where they are reflected back along the aorta as a reflected wave that travels to the finger. Reduced compliance of the elastic arteries shortens the time of return of the ‘reflected wave’, causing a disproportionate elevation in systolic pressure [8]. On pulse wave analysis, such an interaction between the incident pulse wave that travels from the heart to the periphery and the reflected pulse wave from the periphery to the central region can beevaluated and expressed as the “augmentation index”. Besides, increased central arterial stiffness and/or peripheral reflectance is related to an increased propagation speed of waves and proximal shifting of the reflection point in the arterial tree, thereby enhancing the interaction between the incident and reflected waves [9]. Crest time (CT), which is the time interval from foot to peak of a pulse wave, has been found to be a consistent parameter markedly increased in the elderly with arteriosclerosis [10].

R-R interval (RRI) from ECG, which is the time between two successive ventricular depolarizations, has been widely applied in the evaluation of autonomic neural activities [11,12,13]. On the other hand, CT (i.e., when the derivative is equal to zero)has been shown to be a consistent parameter significantly increased in the aged population with arteriosclerosisand could be used for cardiovascular disease classification [14]. While RRI represents changes in electrical activities, CT reflects the actual peripheral microvascular hemodynamic changes. The concept of multiscale allows viewing of the complexity of a set of data from different angles by organizing adjacent data into groups of different sizes (i.e., coarse-grained modeling). The clinical application of multiscale entropy (MSE) in RRI data sets to differentiate patients with congestive heart disease and atrial fibrillation from healthy individuals was first reported in 2002 [15,16,17]. Based on this concept, the use of MSE for CT data analysis has also been found to differentiate among young, aged, and diabetic subjects [18]. Although MSE analysis on single waveform contour parameters (i.e., amplitude, time between systolic and diastolic peaks, CT, and pulse wave velocity) have been used to gain a deeper insight into vascular status [18,19,20], another set of MSE-based indices have been developed after taking into account two synchronized sets of data (i.e., amplitudes from bilateral fingers, time between R wave on ECG and waveform peak from finger, time between R wave and foot point of a waveform) to further reflect vascular health [21,22,23]. The aim of the present study is to test the hypothesis that the application of multiscale cross-approximate entropy (MCAE) analysis in the study of nonlinear coupling behavior of two synchronized time series of different natures (i.e., RRI and CT) could yield information on complexity related to diabetes-associated vascular changes.

## 2. Methods

### 2.1. Study Population

Between July 2009 and March 2012, 95 volunteers were originally enrolled for this study. All diabetic patients were recruited from the diabetes outpatient clinic of the Hualien Hospital, while healthy controls were from a health screening program at the same hospital. Of the 95 subjects, fivewere excluded due to incomplete or unstable waveform data acquisition. The remaining 90 subjects were then divided into three groups, including healthy young subjects (Group 1, age range: 18–40, n = 22), healthy upper middle-aged subjects (Group 2, age range: 41–80, n = 34), type 2 diabetic patients (Group 3, age range: 41–80, n = 34, glycosylated hemoglobin (HbA1c) ≥6.5%) (Table 1) [24]. All healthy subjects had no personal or family history of cardiovascular diseases. Type 2 diabetes was diagnosed by either a fasting blood sugar concentration ≥126 mg/dL or HbA1c ≥6.5%. All diabetic patients received regular treatment and follow-up in the clinic for over two years. The study was approved by Institutional Review Board (IRB) of Hualien Hospital. All subjectswere required to refrain from caffeine-containing beverages and theophylline-containing medications for at least 8 h before each hospital visit. All subjects signed informed consents for the study, completed questionnaires on demographic data and medical histories, and underwent blood sampling before data acquisition.

### 2.2. Study Protocol

A single waveform parameter (i.e., CT) and cardiac electrical parameter (i.e., RRI) were obtained from all subjects. MSE analysis was performed on the acquired data of CT and RRI from scale 1 to scale 6 to obtain multiscale entropy index for CT and RRI [i.e., MEI(CT) and MEI(RRI)], respectively. Cross approximateanalysis on CT and RRI was performed also from scales 1 to 6 to obtain multiscale cross-approximate entropy index (MCEI). Mean values from scales 1 to 3 were defined as small scale (SS), whereas those from scales 4 to 6 were defined as large scale (LS). The associations of the computational parameters thus obtained [i.e., MEI_SS_(CT), MEI_LS_(CT), MEI_SS_(RRI), MEI_LS_(RRI), MCEI_SS_, MCEI_LS_] with the demographic (i.e., age), anthropometric (i.e., body height, body weight, waist circumference, body-mass index), hemodynamic (i.e., systolic and diastolic blood pressures), and serum biochemical (i.e., high- and low-density lipoprotein cholesterol, total cholesterol, and triglyceride) parameters of the three groups of testing subjects were analyzed and compared.

### 2.3. Data Acquisitionand Analysis

All subjects were allowed to rest in a supine position in a quiet, temperature-controlled room at 25 ± 1 °C for 5 min before another 30 min of measurement. Blood pressure was obtained once over the left arm in supine position using an automated oscillometric device (BP3AG1, Microlife, Taibei, Taiwan) with a cuff of appropriate size, followed by collection of data on left index finger waveform using six-channel ECG-pulse wave velocity (PWV) as previously reported [19]. Briefly, the six-channel ECG-PWV system was used for left index finger waveform measurement [7]. Infrared sensors were put on the points of reference simultaneously to acquire data. ECG was obtained using the conventional method. After being processed through an analog-to-digital converter (USB-6009 DAQ, National Instruments, Austin, TX, USA) with a sampling frequency of 500 Hz, the digitized signals were stored in a computer for later analysis. The digital volume pulses (DVPs) were recorded by photoplethysmography, the methodology and the devices of which have been previously reported [19]. We used DVP from the fingertip for waveform contour analysis. The systolic peak and foot point were extracted from the contour of the DVP. The crest time (CT) was the time interval between foot and systolic peak of a pulse wave [10].

#### 2.3.1. Definition of Two Synchronized Physiological Signals: R-R Interval (RRI) and Crest Time (CT)

**Figure 1:**
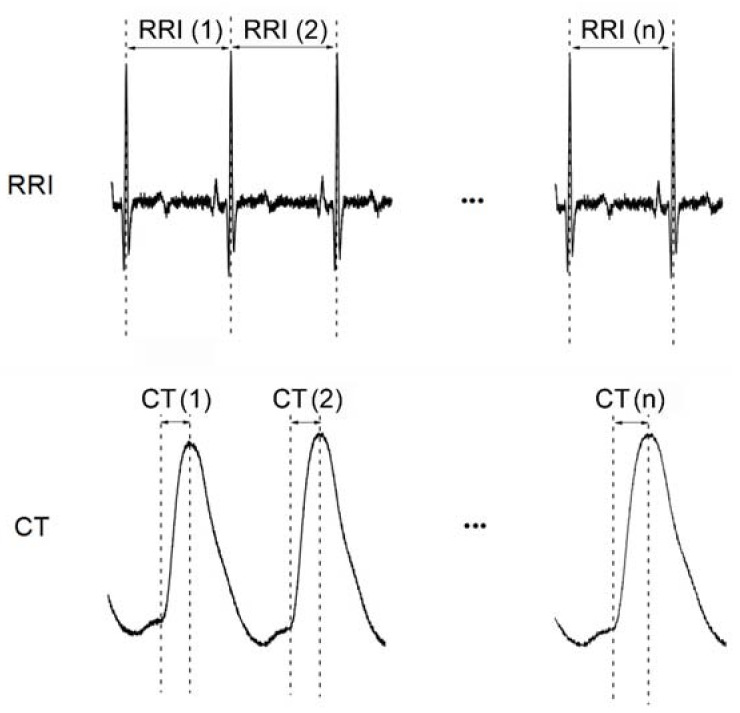
Recording of 1000 consecutive cardiac cycles from electrocardiogram (ECG) and simultaneous arterial waveform signals from photoplethysmography (PPG). RRI: R-R interval; CT: Crest time (i.e., time from foot point to peak of a waveform); RRI(n): RRI during the nth cardiac cycle; CT(n): CT during the nth cardiac cycle.

RRI time series {RRI(*i*)} = {RRI(1), RRI(2), …, RRI(1000)} and CT time series {CT(*j*)} = {CT(1), CT(2), …, CT(1000)} for each participant were obtained from photoplethysmography (PPG) and electrocardiogram (ECG), respectively. One thousand stable consecutive cardiac cycles were obtained from ECG together with the recording of digital waveform signals from PPG within the same period (Figure 1).

#### 2.3.2. MSE and MCAE Analyses

A. Detrending, Normalization, and Coarse-Graining

Acquisition of synchronized RRI and CT signals from 1000 consecutive cardiac cycles gave RRI series {RRI(*i*)}= {RRI(1), RRI(2), …, RRI(1000)} and CT series {CT(*j*)}= {CT(1), CT(2), …, CT(1000)}, respectively, for the purpose of the present study. Due to a trend within physiological signals, non-zero means may be included. Therefore, empirical mode decomposition (EMD) [25] was adopted to deconstruct the {RRI(*i*)}and {CT(*j*)} series, thereby eliminating the trend from the original series. The detrending process consists of decomposing the acquired signals into different intrinsic mode functions (IMFs) which are extracted level by level. First, the highest frequency local oscillations on the corresponding lower frequency part of the data are extracted. The procedure is followed by the extraction of the next level highest-frequency local oscillations of the residual of the data. The process then continues until no complete oscillation can be noted in the residual, which can be considered to be the trend of the original signals in general [26]. Using adaptive decomposition computation of EMD, the number of decomposition levels depends on the length of sampled data [27]. For the present study, a cut-off frequency of around 0.001 Hz was adopted. The {RRI(*i*)} and {CT(*j*)} series was then normalized, as shown in (1). In these equations, SD_RRI(*i*)_ and SD_CT(*j*)_ represent the standard deviations of series {RRI(*i*)}and {CT(*j*)}, respectively. $\bar{\mathrm{RRI}\left(i\right)}$ denotes the mean of the {RRI(*i*)} series, while $\bar{\mathrm{CT}\left(j\right)}$ represents the mean of the {CT(*j*)} series:

(1) $$\left\{{\mathrm{RRI}}^{\prime }\left(i\right)\right\}=\frac{\left\{\mathrm{RRI}\left(i\right)\right\}-\bar{\mathrm{RRI}\left(i\right)}}{{\mathrm{SD}}_{\mathrm{RRI}\left(i\right)}}\;\left\{{\mathrm{CT}}^{\prime }\left(j\right)\right\}=\frac{\left\{\mathrm{CT}\left(j\right)\right\}-\bar{\mathrm{CT}\left(j\right)}}{{\mathrm{SD}}_{\mathrm{CT}\left(j\right)}}$$

The use of a scale factor *τ* (*τ* = 1, 2, 3, …, *n*), which is selected according to a 1-D series of consecutive cycles, is mandatory for multiple analysisto enable the application of a coarse-graining process in order to derive a new series prior to the computation of entropy in each new individual series [15].With this approach, coarse-graining on the normalized 1-D consecutive cycles of the $\left\{{\mathrm{RRI}}^{\prime }\left(i\right)\right\}$ and $\left\{{\mathrm{CT}}^{\prime }\left(j\right)\right\}$ series based on scale factor *τ* can be performed to obtain the series ${\mathrm{RRI}}^{\prime }{}^{\left(\tau \right)}$ and ${\mathrm{CT}}^{\prime }{}^{\left(\tau \right)}$ as shown in (2):

(2) $${\mathrm{RRI}}^{\prime }{\left(u\right)}^{\left(\tau \right)}=\frac{1}{\tau }\overset{u\tau }{\underset{i=\left(u-1\right)\tau +1}{\sum }}{\mathrm{RRI}}^{\prime }\left(i\right),1\leq u\leq \frac{1000}{\tau },\;{\mathrm{CT}}^{\prime }{\left(u\right)}^{\left(\tau \right)}=\frac{1}{\tau }\overset{u\tau }{\underset{j=\left(u-1\right)\tau +1}{\sum }}{\mathrm{CT}}^{\prime }\left(j\right),1\leq u\leq \frac{1000}{\tau },$$

Hence, different multiscale entropy indices and the multiscale cross-approximate entropyindices can be calculated.

B. Computation of MEI for RRI and CT

To assess the complexity of $\left\{{\mathrm{RRI}}^{\prime }\left(i\right)\right\}$ and $\left\{{\mathrm{CT}}^{\prime }\left(j\right)\right\}$ series based on scale factor *τ* series, sample entropy was used for multiscale analysis [28]. The results of sample entropy between scale factors 1 and 3 were defined as small scales, and those between scale factors 4 and 6 were defined as large scales. The mean of sample entropy in small scales of $\left\{{\mathrm{RRI}}^{\prime }\left(i\right)\right\}$ and $\left\{{\mathrm{CT}}^{\prime }\left(j\right)\right\}$ series was defined as MEI_SS_(RRI) vs. MEI_SS_(CT), while the mean of sample entropy in large scales of $\left\{{\mathrm{RRI}}^{\prime }\left(i\right)\right\}$ and $\left\{{\mathrm{CT}}^{\prime }\left(j\right)\right\}$ series was defined as MEI_LS_(RRI) vs. MEI_LS_(CT).To ensure efficiency and accuracy of calculation, the parameters of this study were set at *N* = 1000, *m* = 2, and *r* = 0.15 multiplied by the standard deviation of the time series of $\left\{{\mathrm{RRI}}^{\prime }\left(i\right)\right\}$ and $\left\{{\mathrm{CT}}^{\prime }\left(j\right)\right\}$.

C. Computation of MCEI for Synchronized RRI and CT Signals

Cross-approximate entropy (XApEn) is a refined approximate entropy approach to complexity analysis for the investigation of two sets of synchronized physiological signals [29]. To study the physiological complexity of the acquired signals, XApEn for each time scale was computed using $\left\{{\mathrm{RRI}}^{\prime }{}^{\left(\tau \right)}\right\}$ and $\left\{{\mathrm{CT}}^{\prime }{}^{\left(\tau \right)}\right\}$ time series after the coarse-graining process for Equation (2). The details of the whole algorithm for obtaining the multiscale cross-approximate entropy index (MCEI) are as follows [21,22,23]:

*Step 1*. For a given *m* and two sets of *m*-vectors:

(3) $$x\left(i\right)=\left[{\mathrm{RRI}}^{\prime }{}^{\left(\tau \right)}\left(i\right)\;\;{\mathrm{RRI}}^{\prime }{}^{\left(\tau \right)}\left(i+1\right)\ldots \;\;{\mathrm{RRI}}^{\prime }{}^{\left(\tau \right)}\left(i+m+1\right)\right],i=1,N-m+1\;y\left(j\right)=\left[{\mathrm{CT}}^{\prime }{}^{\left(\tau \right)}\left(j\right)\;\;{\mathrm{CT}}^{\prime }{}^{\left(\tau \right)}\left(j+1\right)\ldots \;\;{\mathrm{CT}}^{\prime \left(\tau \right)}\left(j+m+1\right)\right],j=1,N-m+1$$

*Step 2*. Define the distance between the vectors x(*i*) and y(*j*) as the maximum absolute difference between the correspondingelements in $\left\{{\mathrm{RRI}}^{\prime }\left(i\right)\right\}$ and $\left\{{\mathrm{CT}}^{\prime }\left(j\right)\right\}$ as follows:

(4) $$d\left[x\left(i\right),y\left(j\right)\right]=\underset{k=1,m}{\max}\left[\left|{\mathrm{RRI}}^{\prime }{}^{\left(\tau \right)}\left(i+k-1\right)-{\mathrm{CT}}^{\prime }{}^{\left(\tau \right)}\left(j+k-1\right)\right|\right]$$

*Step 3*. With the given matrix x(*i*) which refers to $\left\{{\mathrm{RRI}}^{\prime }\left(i\right)\right\}$ (where *i* = 1 to *N* − *m* + 1), find the number of time in which *d*[x(*i*), y(*i*)] (where *j* = 1 to *N* − *m* + 1) are smaller than or equal to *r* and the ratio of this number to the total number of m-vectors (*N* − *m* + 1). That is, let $N_{{\mathrm{RRI}}^{\prime }{}^{\left(\tau \right)}{\mathrm{CT}}^{\prime }{}^{\left(\tau \right)}}^{m}\left(i\right)$ equal the number of y(*j*) satisfying the requirement *d*[x(*i*), y(*j*)] ≤*r*; then in (5) $C_{{\mathrm{RRI}}^{\prime }{}^{\left(\tau \right)}{\mathrm{CT}}^{\prime }{}^{\left(\tau \right)}}^{m}$ measures the frequency of the *m*-point $\left\{{\mathrm{CT}}^{\prime }\left(j\right)\right\}$ pattern being similar (within a tolerance of ±*r*) to the *m*-point $\left\{{\mathrm{RRI}}^{\prime }\left(i\right)\right\}$ pattern formed by x(*i*):

(5) $$C_{{\mathrm{RRI}}^{\prime }{}^{\left(\tau \right)}{\mathrm{CT}}^{\prime }{}^{\left(\tau \right)}}^{m}\left(i\right)=\frac{N_{{\mathrm{RRI}}^{\prime }{}^{\left(\tau \right)}{\mathrm{CT}}^{\prime }{}^{\left(\tau \right)}}^{m}\left(i\right)}{N-m+1}$$

*Step4*. Average the logarithm of (5) over *i* to obtain $\emptyset_{\mathrm{RRI},\mathrm{CT}}^{m}\left(r\right)$ as follows:

(6) $$\emptyset_{\mathrm{RRI},\mathrm{CT}}^{m}\left(r\right)=\;\frac{1}{N-m+1}\overset{N-m+1}{\underset{i=1}{\sum }}\ln\left[C_{{\mathrm{RRI}}^{\prime }{}^{\left(\tau \right)}{\mathrm{CT}}^{\prime }{}^{\left(\tau \right)}}^{m}\left(i\right)\right]$$

*Step 5*. Increase *m* by 1 and repeat Steps 1–4 to obtain $C_{{\mathrm{RRI}}^{\prime }{}^{\left(\tau \right)}{\mathrm{CT}}^{\prime }{}^{\left(\tau \right)}}^{m+1}\left(i\right)$ and $\emptyset_{\mathrm{RRI},\mathrm{CT}}^{m+1}\left(r\right)$.

*Step 6*. Finally, for *N*-point data, the estimate is:

(7) $$\mathrm{XApEn}(\mathrm{RRI},\mathrm{CT})=\emptyset_{\mathrm{RRI},\mathrm{CT}}^{m}(r)-\emptyset_{\mathrm{RRI},\mathrm{CT}}^{m+1}(r)$$

where *m* represents the chosen vector dimension, *r* represents a tolerance range, and *N* is the data length. From Pincus’s study, in order to effectively distinguish two data series by cross-approximate entropy, it would be better to set *N* ≥ 1000, *m* ≥ 2, and *r* ≥ 0.1 [30]. To ensure efficiency and accuracy of calculation, the parameters of this study were set at *N* = 1000, *m* = 3, and *r* = 0.6 multiplied by the standard deviation of the time series of {RRI’(*i*)} and {CT’(*j*)}.

Repeat Steps 1–6 to calculate the MCEI in scales 1–6. The values of XApEn (RRI, CT) were obtained from a range of scale factors between 1 and 6. The mean values of XApEn (RRI, CT) between scale factors 1 and 3 were defined as small scales in (8). The mean values of XApEn (RRI, CT) between scale factors 4 and 6 were defined as large scales in (9):

(8) $$\mathrm{MCEIss}\;=\;\frac{1}{3}\overset{3}{\underset{\tau =1}{\sum }}{\mathrm{XApEn}}_{\tau }\left(\mathrm{RRI},\;\mathrm{CT}\right)$$

(9) $$\mathrm{MCEILS}=\;\frac{1}{3}\overset{6}{\underset{\tau =4}{\sum }}{\mathrm{XApEn}}_{\tau }\left(\mathrm{RRI},\;\mathrm{CT}\right)$$

### 2.4. Statistical Analysis

The average values are expressed as mean ± SD. Normality of distribution was tested with one sample Kolmogorov-Smirnov test and the homoscedasticity of variables was verified using the R Language software. The significance of difference in anthropometric, hemodynamic, and computational parameters (i.e., MEI_SS_(RRI), MEI_LS_(RRI), MEI_SS_(CT), MEI_LS_(CT), MCEI_SS_, and MCEI_LS_) among different groups was determined using independent sample *t*-test with Bonferroni correction. The correlation between parameters and risk factors for different groups was compared using Pearson correlation test with Bonferroni correction. For significant parameters acquired through univariate analysis, multivariate regression analysis was used for further verification of the statistical significance. Statistical Package for the Social Science (SPSS, version 14.0 for Windows, SPSS Inc., Chicago, IL, USA) was used for all statistical analyses. Statistical significance was determined using *p* value corrected as shown at the end of each figure and table in the Results section.

## 3. Results

### 3.1. MSE Analysis on Single Waveform Contour Cardiovascular System-Related Parameters (RRI and CT)

**Figure 2:**
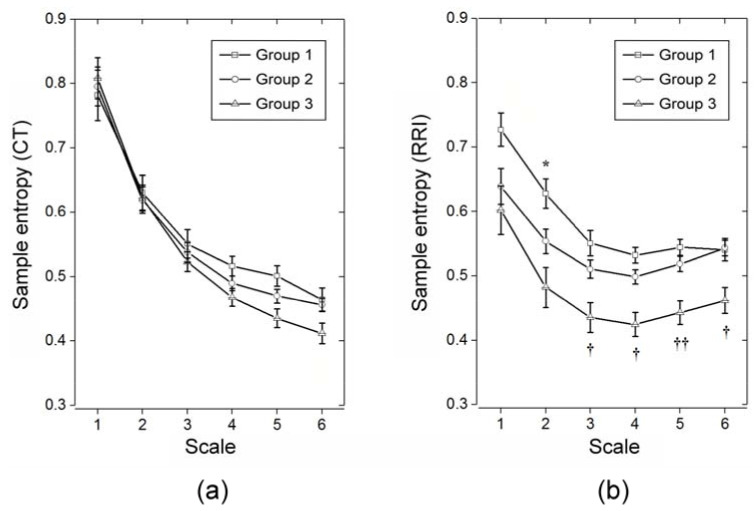
(**a**) Sample entropy of crest time (CT) series of the three groups of testing subjects; (**b**) Sample entropy of R-R interval (RRI) series of the three groups of testing subjects. Values expressed as mean ± standard deviation (SD); Group 1: Healthy young subjects; Group 2: Non-diabetic upper middle-aged subjects; Group 3: Diabetic upper middle-aged subjects; † *p* < 0.017 (*p* corrected): Group 3 vs. Group 1 and Group 2; †† *p* < 0.001: Group 3 vs. Group 1 and Group 2; * *p* < 0.05: Group 1 vs. Group 2 and Group 3.

MSE analysis of CT series of the three groups of participants showed that, although the three groups tended to separate from scale 2 onwards, there was no statistically significant difference among the three groups (Figure 2a, Table 2). For sample entropy of RRI, although the three groups appeared to be separated from scale 1 onwards, significant difference between Group 1 and Group 2 was noted only at scale 2. By contrast, Group 3 had significantly lower sample entropy compared to that of Group 1 and Group 2 from scale 3 onwards (Figure 2b, Table 2).

### 3.2. MultiscaleCross-Approximate Entropy Analysis of Synchronized RRI and CT Time Series

**Figure 3:**
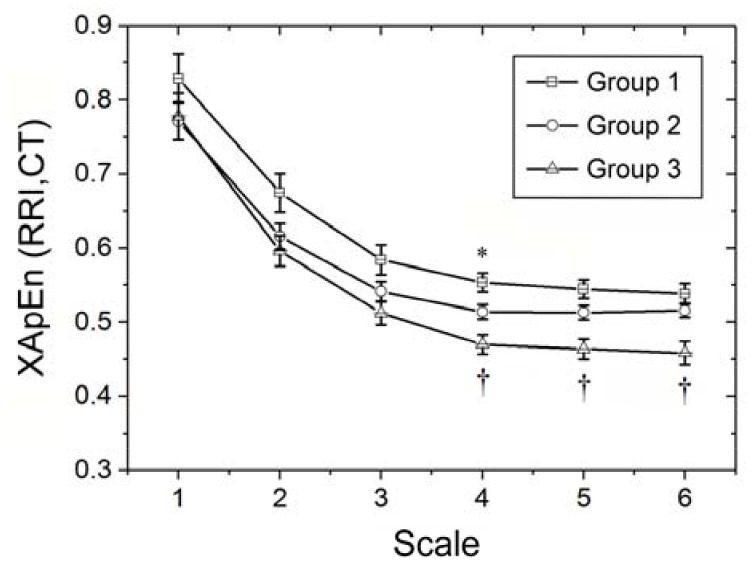
Multiscale cross-approximate entropy analysis of synchronized R-R interval (RRI) and crest time (CT) time series showing changes in cross-approximate entropyof the three groups of testing subjects with time scale 1 to 6. Group 1: Healthy young subjects; Group 2: Non-diabetic upper middle-aged subjects; Group 3: Diabetic upper middle-aged subjects; * *p* < 0.017 (*p* corrected): Group 1 vs. Group 2; † *p* < 0.017: Group 2 vs. Group 3.

MCAE analysis of synchronized RRI and CT series of the testing subjects using cross-approximate entropy demonstrated a unanimous decrease in all groups from scale 1 to scale 4 (Figure 3). Significant differentiation among the three groups was noted at scales 4 (*p* < 0.017). While Group 1 had the highest cross-approximate entropy at scale 4, Group 3 had the lowest cross-approximate entropy at scale 4, 5, and 6 (Figure 3, Table 2).

Comparison of multiscale entropy indices for crest time and R-R interval at different time scales [i.e., MEI(CT), MEI(RRI), and MCEI(CT,RRI)] among the testing subjects showed no significant difference among the three groups in MEI for CT (Table 2). On the other hand, small-scale MEI for RRI [i.e., MEI_SS_(RRI)] successfully differentiated Group 2 from Group 1 at time scale 2 (Figure 2a). As a whole, MEI_SS_(RRI) was significantly lower in Group 2 than that in Group 1 (Table 2). By contrast, MEI for RRI of Group 3 was significantly lower than that in Group 2 at scale 3, 4, 5, and 6. Consistently, large-scale MEI for RRI [i.e., MEI_LS_(RRI)] was significantly lower in Group 3 than that in Group 2 (*p* < 0.001). As for cross-approximate entropy index for synchronized RRI and CT time series [i.e., MCEI(RRI,CT)], it is interesting to find that there were significant differences among the three groups at scale 4. On the other hand, large-scale MCEI [i.e., MCEI_LS_(RRI,CT)] was significantly lower in Group 3 than that in Groups 1 and 2 (Table 2).

### 3.3. Correlations of Different Multiscale Entropy Indices with Demographic, Anthropometric, Hemodynamic, and Serum Biochemical Parameters in the Testing Subjects

To study theassociations of different multiscale entropy indices [i.e., MEI_SS_(CT), MEI_LS_(CT), MEI_SS_(RRI), MEI_LS_(RRI), MCEI_LS_(RRI,CT), MCEI_SS_(RRI,CT)]with demographic, anthropometric, hemodynamic, and serum biochemical parameters in non-diabetic subjects, healthy young individuals (Group 1) and upper middle-aged non-diabetic subjects (Group 2) were investigated (Table 3). Total cholesterol levelwas found to be negatively associated with MEI_LS_(RRI) (*p* = 0.003).

To investigate the correlations of different multiscale entropy indices with demographic, anthropometric, hemodynamic, and serum biochemical parameters inupper middle-aged subjects, upper middle-aged non-diabetic (Group 2) and diabetic (Group 3) subjects were studied together (Table 4). Body weight was found to be negatively associated with MCEI_LS_(RRI,CT) (*p* < 0.017). Besides, waist circumference was negatively related to MEI_LS_(CT), MEI_LS_(RRI), and MCEI_LS_(RRI,CT) (all *p* < 0.017). By contrast, pulse pressure was positively correlated with MCEI_SS_(RRI,CT) (*p* < 0.017). On the other hand, MEI_LS_(RRI) was negatively associated with glycated hemoglobin (HbA1c) level and fasting blood sugar concentration (both *p* < 0.017) (Table 4).

When both age and diabetes were taken into account by taking all three groups of testing subjects into consideration (Table 5), MEI_SS_(RRI), MEI_LS_(RRI), and MCEI_LS_(RRI,CT) were found to be negatively associated with age in a highly significant way (all *p* < 0.005). Moreover, negative correlations were also noted between body weight and MEI_SS_(CT) (*p* < 0.017). While waist circumference was negatively correlated with all multiscale entropy parameters except MCEI_SS_(RRI,CT), body-mass index was negatively associated with MEI_SS_(RRI), MEI_LS_(RRI), and MCEI_LS_(RRI,CT) (all *p* < 0.017). On the other hand, glycated hemoglobin levelwas negatively associated with MEI_LS_(CT), MEI_SS_(RRI),MEI_LS_(RRI), andMCEI_LS_(RRI,CT), while fasting blood sugar levels were negatively associated with MEI_SS_(RRI), MEI_LS_(RRI),and MCEI_LS_(RRI,CT) (all *p* < 0.017).Both sugar control parameters were highly significantly correlated withMEI_LS_(RRI) and MCEI_LS_(RRI,CT) in a negative fashion (all *p* < 0.005).

### 3.4. Multivariate Analysis for MEI_LS_(CT), MEI_LS_(RRI), and MCEI_LS_(RRI,CT)

The three multiscale entropy indices found to be significantly associated with the demographic, anthropometric, hemodynamic, and serum biochemical parameters of the testing subjects using Pearson correlation test (Table 5) were MEI_LS_(CT), MEI_LS_(RRI), and MCEI_LS_(RRI,CT) for which multivariate analysis was performed. The results showed significant associations of MEI_LS_(RRI), and MCEI_LS_(RRI,CT) with age and glycated hemoglobin level in all subjects as a whole without focusing on the effects of age and diabetes (all *p* < 0.05) (Table 6).

## 4. Discussion

Taking into consideration the clinical implications of non-invasive indices including digital volume pulse (DVP) from photoplethysmography [6,7] and R-R interval (RRI) from electrocardiogram [11], the present study tested the value of a multiscale entropy index utilizing two synchronized sets of non-invasively acquired physiological data [i.e., MCEI(RRI,CT)] in discerning the adverse impacts of systemic conditions (i.e., age and diabetes) on vascular health in a clinical setting. Our results demonstrated a unique property of the novel index in identifying diabetes-associated changes in vascular condition in human subjects.

In 2002, Peng et al. first described the application of multiscale entropy in the analysis of R-R interval from electrocardiogram to differentiate healthy individuals from those with congestive heart failure and atrial fibrillation who were found to have significantly reduced signal complexity [15]. To evaluate the impact of age and a systemic disease (i.e., diabetes) on vascular health, the current study attempted to adopt different multiscale entropy indices to identify the one that could best reflect the influences of such systemic conditions. Previous studies have demonstrated that small-scale multiscale entropy represents autonomic nervous activity, whereas large-scale multiscale entropy reflects vascular regulatory function [28,31]. In the present study, MEI_SS_(RRI) identified age-related vascular changes by significantly differentiating between young (Group 1) and healthy upper middle-aged (Group 2) subjects (*p* = 0.015) (Table 2). Since small scales (i.e., scale 1 to 3) reflects autonomic nervous control of the cardiovascular system in the present setting, the finding of reduced MEI_SS_(RRI) in Group 2 suggests significantly elevated resting sympathetic tone in Group 2 compared to that in Group 1. The results is supported by a previous study showing an increase in resting sympathetic outflow with age [32]. In addition, our results demonstrated consistent and significant reductions of MEI_LS_(RRI) in upper middle-aged individuals with diabetes (Group 3) compared to those without (Group 2) (Table 2), highlighting its ability to identify diabetes-associated vascular changes. The finding is consistent with the fact that diabetes impairs vascular structural integrity [33]. On the other hand, MEI_LS_(RRI) failed to detect age-related vascular changes (i.e., between Group 1 and Group 2), underscoring its limitation in this aspect.

The physiological significance of the application of multiscale cross-approximate entropy (MCAE) analysis in this study is the identification of subtle differences in age- and diabetes-associated vascular changes by comparing the degree of nonlinear coupling between two related synchronized time series. While RRI stands for the electrical component of cardiovascular activities, CT represents the mechanical component. It has been documented that, although age and diabetes are both systemic conditions adversely affect vascular integrity, the mechanisms are different. While aging is known to cause arterial medial degeneration involving increase in collagen and calcium deposits as well as elastin lamellae fragmentation resulting from upregulation of proteolytic enzymes and possible repetitive cyclic stress on the arterial wall over a life span [34,35], diabetes mellitus has been shown to be associated with the generation of advanced glycation end-products (AGEs) that give raise to collagen crosslinking in the arterial medial layer and contributes to arterial stiffness [33].

Analysis of the correlations of different multiscale entropy indices with demographic, anthropometric, hemodynamic, and serum biochemical parameters in non-diabetic subjects showed a significant negative association of MEI_LS_(RRI) with serum cholesterol level (Table 3). As large-scale entropy represents vascular regulatory function, the finding is consistent with that of a previous study demonstrating that the cellular and molecular mechanisms underlying hypercholesterolemia could contribute to an imbalance between phosphorylation and dephosphorylation of lipid and protein kinase, thereby modulating vascular endothelial L-arginine/nitric oxide synthetase (eNOS) and produce vascular endothelium dysfunction [36]. The significant negative correlations ofbody weightwith MCEI_LS_(RRI,CT) as well as wrist circumference with MEI_LS_(CT), MEI_LS_(RRI), and MCEI_LS_(RRI,CT) in upper middle-aged subjects with and without diabetes (Group 3 and Group 2) (Table 4) imply an association of increased anthropometric parameters with impaired vascular function in the aged subjects. The significant negative associations of MEI_LS_(RRI) with fasting blood sugar andglycated hemoglobin levels (Table 4) further highlight the adverse impact of diabetes on vascular endothelial function in both acute [37] and chronic [38] hyperglycemia, respectively.

When all subjects were taken into account (Table 5), MEI_SS_(RRI), MEI_LS_(RRI), MCEI_LS_ were all negatively associated with age in a highly significant manner (*p* < 0.005). The results underscore the sensitivity of MEI(RRI) in discerning age-related changes in both autonomic nervous control and function of the cardiovascular system, while MCEI seems sensitive to age-related alterations in cardiovascular function. On the other hand, negative associations of different multiscale entropy indices with anthropometric parameters (i.e., waist circumference, and body-mass index) in general reflect the sensitivity of these indices in identifying anthropometric anomalies contributing to adverse changes in cardiovascular function. In addition, the significant negative associations of MEI_SS_(RRI), MEI_LS_(RRI), and MCEI_LS_(RRI,CT) with the parameters of acute (i.e., fasting blood sugar level) and chronic (i.e., glycated hemoglobin concentration) blood sugar control indicate the sensitivity of MEI(RRI) in reflecting blood sugar-related changes in both autonomic nervous control and function of the cardiovascular system, whereas MEI(CT) and MCEI appear to be indicators of hyperglycemia-related vascular changes. To further elucidate the significance of these parameters, multivariate linear regression analysis demonstrated significant associations of MEI_LS_(RRI), and MCEI_LS_(RRI,CT) with age and glycated hemoglobin level (all *p* < 0.05) (Table 6).

Like MEI_LS_(RRI), the successful differentiation between upper-middle aged subjects with (Group 3) and without (Group 2) diabetes using MCEI_LS_(RRI,CT) (Table 2) highlights the ability of this index to discern diabetes-related changes in vascular regulatory function. In addition, MCEI(RRI,CT) was the only multiscale entropy index that successfully differentiated among the three groups at scale 4 in the present study (Figure 3). This index was also found to be negatively related to anthropometric parameters (i.e., wrist circumference, body-mass index) and fasting blood sugar (Table 5), underlining its association with metabolic syndrome.

There are several limitations of the present study. First, since the harvesting of PPG signals from the finger is prone to interference from body motion such as respiratory movement, detrending with EMD was performed before standardization of the two times series. Second, there were eight patients in Group 3 with established diagnosis of hypertension under medical control. Since those patients have different combinations of anti-hypertensives which also changed during different periods of treatment (e.g., from calcium channel blocker to angiotensin-converting-enzyme inhibitor), it was difficult to determine who should be excluded from the study. Therefore, the potential influence of anti-hypertensive agents on their spontaneous cardiac activity cannot be excluded.Third, although it has been reported that cross-sample entropy could avoid some potential problems related to XApEn [39], cross-sample entropy analysis was not performed in the present study for comparison.

## 5. Conclusions

The present study demonstrated the successful application of multiscale cross-approximate entropy (MCAE) analysis in the study of two synchronized time series of different natures to yield additional information on complexity related to diabetes-associated vascular changes. Our findings showed that MCEI_LS_ could serve as a novel non-invasive biomarker for discerning diabetes-related changes in the cardiovascular system, which is of clinical importance in preventive medicine. The results of the current study successfully identified the risk factors for cardiovascular diseases by comparing the nonlinear coupling behavior of two cardiovascular system-related synchronized time series of different natures. It is anticipated that the risk factors of diseases of other organ systems could be identified with this approach through the analysis ofnonlinear coupling of different synchronized physiological signals pertinent to different organ systems.

## Figures and Tables

**Table 1:** Demographic, anthropometric, hemodynamic, and serum biochemical parameters of the testing subjects.

Parameters	Group 1 (n = 22)	Group 2 (n = 34)	Group 3 (n = 34)
Male/Female	13/9	10/24	22/12
Age (years)	28.68 ± 6.34	56.21 ± 10.72 **	60.71 ± 8.46
Body weight (kg)	68.27 ± 15.89	61.73 ± 10.55	73.88 ± 14.86 ††
WC(cm)	82.30 ± 13.53	80.79 ± 9.43	95.00 ± 11.56 ††
BMI (kg/m^2^)	23.60 ± 4.48	23.72 ± 3.54	27.92 ± 4.70 ††
SBP (mmHg)	117.46 ± 10.94	118.97 ± 16.60	127.38 ± 17.14 ††
DBP (mmHg)	73.91 ± 7.02	72.97 ± 9.03	76.06 ± 10.16
PP (mmHg)	43.55 ± 7.65	46.00 ± 11.12	51.32 ± 13.84
HDL (mg/dL)	46.46 ± 15.34	55.27 ± 19.34	40.21 ± 13.13 ††
LDL (mg/dL)	124.86 ± 41.11	157.88 ± 43.48 *	148.62 ± 47.39
Cholesterol (mg/dL)	174.64 ± 56.33	165.44 ± 94.19	154.94 ± 53.51
Triglyceride (mg/dL)	79.64 ± 36.31	102.03 ± 30.99 *	117.59 ± 45.06 †
HbA1c(%)	5.51 ± 0.34	5.87 ± 0.40 **	8.14 ± 1.27 ††
PWV_finger_(m/sec)	4.48 ± 0.87	4.88 ± 0.49	5.93 ± 0.58 †

Values are expressed as mean ± SD; Group 1: Healthy young subjects; Group 2: Healthy upper middle-aged subjects; Group 3: Type 2 diabetic patients; BMI: Body mass index; SBP: Systolic blood pressure; DBP: Diastolic blood pressure; PP: Pulse pressure; WC: Waist circumference; HbA1c: Glycated hemoglobin; PWV_finger_: Left index finger pulse wave velocity [19]; * *p* < 0.017 (*p* corrected): Group 1 vs. Group2; ** *p* < 0.001: Group 1 vs. Group 2; † *p* < 0.017: Group 2 vs. Group 3; †† *p* < 0.001: Group 2 vs. Group 3.

**Table 2:** Multiscale entropy indices for crest time and R-R interval at different time scales in three groups of testing subjects.

Parameters	Group 1 (n = 22)	Group 2 (n = 34)	Group 3 (n = 34)
MEI_SS_(CT)	0.65 ± 0.13	0.65 ± 0.12	0.65 ± 0.13
MEI_LS_(CT)	0.49 ± 0.07	0.47 ± 0.06	0.44 ± 0.08
MEI_SS_(RRI)	0.64 ± 0.08	0.58 ± 0.11 *	0.51 ± 0.17
MEI_LS_(RRI)	0.54 ± 0.06	0.52 ± 0.07	0.44 ± 0.11 ††
MCEI_SS_(RRI,CT)	0.70 ± 0.11	0.64 ± 0.10	0.63 ± 0.12
MCEI_LS_(RRI,CT)	0.55 ± 0.06	0.51 ± 0.06	0.46 ± 0.08 †

Values are expressed as mean ± SD; Group 1: Healthy young subjects; Group 2: Non-diabetic upper middle-aged subjects; Group 3: Diabetic upper middle-aged subjects. MEI: Multiscale entropy index; CT: Crest time; RRI: R-R interval; MEI_SS_(CT): Small-scale multiscale entropy index for crest time (i.e., average MEI for CT series of time scale 1, 2, and 3); MEI_LS_(CT): Large-scale multiscale entropy index for crest time (i.e., average MEI for CT series of time scale 4, 5, and 6); MEI_SS_(RRI): Small-scale multiscale entropy index for R-R interval (i.e., average MEI forRRI series at time scale 1, 2, and 3); MEI_LS_(RRI): Large-scale multiscale entropy index for R-R interval (i.e., average MEI for RRI series at time scale 4, 5, and 6); MCEI_SS_(RRI,CT): Small-scale multiscale cross-approximate entropy index (i.e., average MCEI for synchronized RRI and CT series at time scale 1, 2, and 3); MCEI_LS_(RRI,CT): Large-scale multiscale cross-approximate entropy index (i.e., average MCEI for synchronized RRI and CT series at time scale 4, 5, and 6); * *p* < 0.017 (*p* corrected): Group 1 vs. Group 2; † *p* < 0.017: Group 2 vs. Group 3; †† *p* < 0.001: Group 2 vs. Group 3.

**Table 3:** Correlations of different multiscale entropy indices with demographic, anthropometric, hemodynamic, and serum biochemical parameters in young healthy individuals (Group 1) and non-diabetic upper middle-aged subjects (Group 2) (n = 56).

	MEI_SS_(CT)		MEI_LS_(CT)		MEI_SS_(RRI)		MEI_LS_(RRI)		MCEI_SS_		MCEI_LS_	
	*p*	*r*	*p*	*r*	*p*	*r*	*p*	*r*	*p*	*r*	*p*	*r*
**Age (years)**	0.736	0.046	0.649	−0.062	0.102	−0.221	0.385	−0.118	0.408	−0.113	0.125	−0.207
**BH (cm)**	0.254	−0.155	0.265	−0.151	0.819	−0.031	0.227	−0.164	0.537	0.084	0.491	−0.094
**BW (kg)**	0.081	−0.236	0.996	0.001	0.152	−0.194	0.327	−0.134	0.921	−0.014	0.771	−0.040
**WC (cm)**	0.064	−0.250	0.974	−0.005	0.180	−0.182	0.301	−0.141	0.584	−0.075	0.506	−0.091
**BMI (kg/m^2^)**	0.161	−0.190	0.362	0.124	0.074	−0.241	0.705	−0.052	0.449	−0.103	0.874	0.022
**SBP (mmHg)**	0.209	0.171	0.259	0.153	0.398	0.115	0.332	0.132	0.113	0.214	0.107	0.218
**DBP (mmHg)**	0.742	0.045	0.183	0.181	0.740	0.045	0.877	−0.021	0.533	0.085	0.525	0.087
**PP (mmHg)**	0.115	0.213	0.584	0.075	0.334	0.131	0.117	0.212	0.070	0.244	0.065	0.248
**HDL (mg/dL)**	0.802	0.034	0.254	−0.153	0.819	−0.031	0.915	−0.015	0.418	−0.110	0.254	−0.155
**LDL (mg/dL)**	0.515	0.089	0.590	−0.074	0.051	−0.263	0.239	−0.160	0.927	−0.012	0.259	−0.153
**Cholesterol (mg/dL)**	0.403	−0.114	0.740	−0.045	0.067	−0.247	0.003*	−0.394	0.058	−0.255	0.020	−0.311
**Triglyceride (mg/dL)**	0.958	−0.007	0.857	0.025	0.681	−0.056	0.365	−0.123	0.910	0.016	0.451	−0.103
**HbA1c (%)**	0.332	0.132	0.947	0.009	0.226	−0.164	0.911	0.015	0.958	0.007	0.641	0.064
**FBS (mg/dL)**	0.626	0.066	0.606	0.070	0.683	0.056	0.315	−0.137	0.759	−0.042	0.451	−0.103

Values are expressed as mean ± SD; Group 1: Healthy young subjects; Group 2: Non-diabetic upper middle-aged subjects. MEI: Multiscale entropy index; CT: Crest time; RRI: R-R interval; MEI_τ=n_(CT): Multiscale entropy index for crest time series at time scale n; MEI_SS_(CT): Small-scale multiscale entropy index for crest time (i.e., average MEI for CT series of time scale 1, 2, and 3); MEI_LS_(CT): Large-scale multiscale entropy index for crest time (i.e., average MEI for CT series of time scale 4, 5, and 6); MEI_τ=n_(RRI): Multiscale entropy index for R-R interval series at time scale n; MEI_SS_(RRI): Small-scale multiscale entropy index for R-R interval (i.e., average MEI forRRI series at time scale 1, 2, and 3); MEI_LS_(RRI): Large-scale multiscale entropy index for R-R interval (i.e., average MEI for RRI series at time scale 4, 5, and 6); MCEI_τ=n_(RRI,CT): Multiscale cross-approximate entropy index for synchronized R-R interval and crest time series at time scale n;MCEI_SS_(RRI,CT): Small-scale multiscale cross-approximate entropy index (i.e., average MCEI for synchronized RRI and CT series at time scale 1, 2, and 3); MCEI_LS_(RRI,CT): Large-scale multiscale cross-approximate entropy index (i.e., average MCEI for synchronized RRI and CT series at time scale 4, 5, and 6); * *p* < 0.017 (*p* corrected).

**Table 4:** Correlations of different multiscale entropy indices with demographic, anthropometric, hemodynamic, and serum biochemical parameters in upper middle-aged non-diabetic subjects (Group 2) and diabetic patients (Group 3) (n = 68).

	MEI_SS_(CT)		MEI_LS_(CT)		MEI_SS_(RRI)		MEI_LS_(RRI)		MCEI_SS_		MCEI_LS_	
	*p*	*r*	*p*	*r*	*p*	*r*	*p*	*r*	*p*	*r*	*p*	*r*
**Age (years)**	0.409	0.102	0.778	−0.035	0.395	−0.105	0.029	−0.264	0.725	0.043	0.272	−0.135
**BH (cm)**	0.079	−0.214	0.248	−0.142	0.656	−0.055	0.793	−0.032	0.070	−0.221	0.222	−0.150
**BW (kg)**	0.044	−0.245	0.040	−0.250	0.086	−0.210	0.057	−0.232	0.032	−0.260	0.017 *	−0.288
**WC (cm)**	0.031	−0.262	0.009 *	−0.314	0.063	−0.227	0.014 *	−0.298	0.042	−0.248	0.004 *	−0.343
**BMI (kg/m^2^)**	0.190	−0.161	0.105	−0.198	0.067	−0.223	0.043	−0.246	0.139	−0.181	0.047	−0.242
**SBP (mmHg)**	0.136	0.183	0.580	0.068	0.729	0.043	0.416	−0.100	0.165	0.170	0.875	0.019
**DBP (mmHg)**	0.757	−0.038	0.924	0.012	0.213	−0.153	0.116	−0.192	0.540	−0.076	0.697	−0.048
**PP (mmHg)**	0.022	0.276	0.498	0.084	0.156	0.174	0.936	0.010	0.017 *	0.288	0.611	0.063
**HDL (mg/dL)**	0.923	0.012	0.267	0.136	0.400	0.104	0.067	0.224	0.636	0.058	0.077	0.216
**LDL (mg/dL)**	0.555	0.073	0.833	0.026	0.187	−0.162	0.829	−0.027	0.869	0.020	0.482	0.087
**Cholesterol (mg/dL)**	0.464	−0.090	0.905	0.015	0.179	−0.165	0.087	−0.209	0.263	−0.138	0.259	−0.142
**Triglyceride (mg/dL)**	0.671	0.052	0.434	0.096	0.762	0.037	0.760	0.038	0.531	0.077	0.383	0.107
**HbA1c (%)**	0.808	0.030	0.102	−0.200	0.225	−0.149	0.015 *	-0.294	0.875	0.020	0.077	−0.216
**FBS (mg/dL)**	0.778	0.035	0.092	−0.206	0.148	−0.177	0.005 *	-0.335	0.955	0.007	0.043	−0.246

Values are expressed as mean ± SD; Group 2: Non-diabetic upper middle-aged subjects; Group 3: Diabetic upper middle-aged subjects. MEI: Multiscale entropy index; CT: Crest time; RRI: R-R interval; MEI_τ=n_(CT): Multiscale entropy index for crest time series at time scale n; MEI_SS_(CT): Small-scale multiscale entropy index for crest time (i.e., average MEI for CT series of time scale 1, 2, and 3); MEI_LS_(CT): Large-scale multiscale entropy index for crest time (i.e., average MEI for CT series of time scale 4, 5, and 6); MEI_τ=n_(RRI): Multiscale entropy index for R-R interval series at time scale n; MEI_SS_(RRI): Small-scale multiscale entropy index for R-R interval (i.e., average MEI forRRI series at time scale 1, 2, and 3); MEI_LS_(RRI): Large-scale multiscale entropy index for R-R interval (i.e., average MEI for RRI series at time scale 4, 5, and 6); MCEI_τ=n_(RRI,CT): Multiscale cross-approximate entropy index for synchronized R-R interval and crest time series at time scale n;MCEI_SS_(RRI,CT): Small-scale multiscale cross-approximate entropy index (i.e., average MCEI for synchronized RRI and CT series at time scale 1, 2, and 3); MCEI_LS_(RRI,CT): Large-scale multiscale cross-approximate entropy index (i.e., average MCEI for synchronized RRI and CT series at time scale 4, 5, and 6); * *p* < 0.017 (*p* corrected).

**Table 5:** Correlations of different multiscale entropy indices with demographic, anthropometric, hemodynamic, and serum biochemical parameters in healthy young adults (Group 1), upper middle-aged non-diabetic subjects (Group 2) and diabetic patients (Group 3) (n = 90).

	MEI_SS_(CT)		MEI_LS_(CT)		MEI_SS_(RRI)		MEI_LS_(RRI)		MCEI_SS_		MCEI_LS_
	*p*	*r*	*p*	*r*	*p*	*r*	*p*	*r*	*p*	*r*	*r*
**Age (years)**	0.779	0.030	0.097	−0.176	0.003 *	−0.310	0.001 *	−0.332	0.135	−0.159	−0.334
**BH (cm)**	0.037	−0.221	0.272	−0.117	0.838	0.022	0.743	0.035	0.696	−0.042	−0.013
**BW (kg)**	0.006*	−0.286	0.053	−0.204	0.040	−0.217	0.046	−0.211	0.089	−0.180	−0.237
**WC (cm)**	0.008 *	−0.280	0.012 *	−0.263	0.007 *	−0.284	0.004 *	−0.300	0.038	−0.219	−0.327
**BMI (kg/m^2^)**	0.065	−0.195	0.137	−0.158	0.008 *	−0.276	0.010 *	−0.269	0.073	−0.190	−0.261
**SBP (mmHg)**	0.343	0.101	0.894	0.014	0.744	−0.035	0.309	−0.108	0.368	0.096	−0.016
**DBP (mmHg)**	0.728	−0.037	0.907	0.013	0.143	−0.156	0.078	−0.187	0.538	−0.066	−0.073
**PP (mmHg)**	0.119	0.165	0.927	0.010	0.504	0.071	0.966	−0.005	0.089	0.180	0.034
**HDL (mg/dL)**	0.653	0.048	0.247	0.123	0.340	0.102	0.044	0.213	0.963	0.005	0.172
**LDL (mg/dL)**	0.555	0.063	0.449	−0.081	0.043	−0.213	0.229	−0.128	0.895	−0.014	−0.074
**Cholesterol (mg/dL)**	0.495	−0.073	0.897	−0.014	0.316	−0.107	0.114	−0.168	0.241	−0.125	−0.139
**Triglyceride (mg/dL)**	0.815	0.025	0.983	−0.002	0.467	−0.078	0.333	−0.103	0.885	0.015	−0.053
**HbA1c (%)**	0.842	0.021	0.013 *	−0.261	0.013 *	−0.261	0.001 **	−0.354	0.430	−0.084	−0.306
**FBS (mg/dL)**	0.846	0.021	0.023	−0.239	0.012 *	−0.263	<0.001 **	−0.384	0.391	−0.091	−0.322

Values are expressed as mean ± SD; Group 1: Healthy young subjects; Group 2: Non-diabetic upper middle-aged subjects; Group 3: Diabetic upper middle-aged subjects. MEI: Multiscale entropy index; CT: Crest time; RRI: R-R interval; MEI_τ=n_(CT): Multiscale entropy index for crest time series at time scale n; MEI_SS_(CT): Small-scale multiscale entropy index for crest time (i.e., average MEI for CT series of time scale 1, 2, and 3); MEI_LS_(CT): Large-scale multiscale entropy index for crest time (i.e., average MEI for CT series of time scale 4, 5, and 6); MEI_τ=n_(RRI): Multiscale entropy index for R-R interval series at time scale n; MEI_SS_(RRI): Small-scale multiscale entropy index for R-R interval (i.e., average MEI forRRI series at time scale 1, 2, and 3); MEI_LS_(RRI): Large-scale multiscale entropy index for R-R interval (i.e., average MEI for RRI series at time scale 4, 5, and 6); MCEI_τ=n_(RRI,CT): Multiscale cross-approximate entropy index for synchronized R-R interval and crest time series at time scale n;MCEI_SS_(RRI,CT): Small-scale multiscale cross-approximate entropy index (i.e., average MCEI for synchronized RRI and CT series at time scale 1, 2, and 3); MCEI_LS_(RRI,CT): Large-scale multiscale cross-approximate entropy index (i.e., average MCEI for synchronized RRI and CT series at time scale 4, 5, and 6); * *p* < 0.017 (*p* corrected), ** *p* < 0.001.

**Table 6:** Multivariate linear regression analysis for MEI_LS_(CT), MEI_LS_(RRI), and MCEI_LS_(RRI,CT) for all subjects (n = 90).

	MEI_LS_(CT)			MEI_LS_(RRI)			MCEI_LS_(RRI,CT)		
	B-Coef	SE	*p*	B-Coef	SE	*p*	B-Coef	SE	*p*
Variable									
Age (year)	0.000	0.001	0.569	−0.001	0.001	0.040	−0.001	0.001	0.022
HbA1c(%)	−0.012	0.006	0.041	−0.018	0.007	0.012	−0.012	0.006	0.041
**B0**	0.563	0.039	<0.001	0.686	0.046	<0.001	0.644	0.036	<0.001

B-Coef:Regression coefficient; SE:Standardized regression coefficient; HbA1c: Glycated hemoglobin.

## References

[1] Lennon R.P., Claussen K.A., Kuersteiner K.A. (2018). State of the Heart: An Overview of the Disease Burden of Cardiovascular Disease from an Epidemiologic Perspective. Prim. Care.

[2] Wu H.T., Lin B.Y., Yang C.C., Ou Y.N., Sun C.K. (2017). Assessment of Vascular Health With Photoplethysmographic Waveforms From the Fingertip. IEEE J. Biomed. Health Inform..

[3] Freis E.D., Heath W.C., Luchsinger P.C., Snell R.E. (1966). Changes in the carotid pulse which occur with age and hypertension. Am. Heart J..

[4] Endresen J., Gamble A., Hill D.W. (1975). A comparison of two methods for the computer analysis of arterial blood pressure waveforms. Eur. J. Intensive Care Med..

[5] Kelly R., Hayward C., Avolio A., O’Rourke M. (1989). Noninvasive determination of age-related changes in the human arterial pulse. Circulation.

[6] Su F., Li Z., Sun X., Han N., Wang L., Luo X. (2014). The pulse wave analysis of normal pregnancy: Investigating the gestational effects on photoplethysmographic signals. Biomed. Mater. Eng..

[7] Wu H.T., Lee K.W., Pan W.Y., Liu A.B., Sun C.K. (2017). Difference in bilateral digital volume pulse as a novel non-invasive approach to assessing arteriosclerosis in aged and diabetic subjects: A preliminary study. Diabetes Vasc. Dis. Res..

[8] Elgendi M. (2012). On the analysis of fingertip photoplethysmogram signals. Curr. Cardiol. Rev..

[9] Tomiyama H., Yamashina A. (2010). Non-invasive vascular function tests: Their pathophysiological background and clinical application. Circ. J..

[10] Hsu P.C., Wu H.T., Sun C.K. (2018). Assessment of Subtle Changes in Diabetes-Associated Arteriosclerosis using Photoplethysmographic Pulse Wave from Index Finger. J. Med.Syst..

[11] Pomeranz B., Macaulay R.J., Caudill M.A., Kutz I., Adam D., Gordon D., Kilborn K.M., Barger A.C., Shannon D.C., Cohen R.J. (1985). Assessment of autonomic function in humans by heart rate spectral analysis. Am. J. Physiol..

[12] Huynh L.N., Truong Q.D.K., Vo V.T., Nguyen B.T., Truong Q.D.K., Tran H.L.P. (2013). FIDELITY: Fuzzy inferential diagnostic engine for on-Line support to physicians. 4th International Conference on Biomedical Engineering in Vietnam, IFMBE Proceedings.

[13] Villecco F., Pellegrino A. (2017). Evaluation of uncertainties in the design process of complex mechanical systems. Entropy.

[14] Alty S.R., Angarita-Jaimes N., Millasseau S.C., Chowienczyk P.J. (2007). Predicting arterial stiffness from the digital volume pulse waveform. IEEE Trans. Biomed. Eng..

[15] Costa M., Goldberger A.L., Peng C.K. (2002). Multiscale entropy to distinguish physiologic and synthetic RR time series. Comput. Cardiol..

[16] Gao Y., Villecco F., Li M., Song W. (2017). Multi-scale permutation entropy based on improved LMD and HMM for rolling bearing diagnosis. Entropy.

[17] Villecco F., Pellegrino A. (2017). Entropic measure of epistemic uncertainties in multibody system models by axiomatic design. Entropy.

[18] Liu A.B., Wu H.T., Liu C.W., Liu C.C., Tang C.J., Tsai I.T., Sun C.K. (2015). Application of multiscale entropy in arterial waveform contour analysis in healthy and diabetic subjects. Med. Biol. Eng. Comput..

[19] Wu H.T., Hsu P.C., Lin C.F., Wang H.J., Sun C.K., Liu A.B., Lo M.T., Tang C.J. (2011). Multiscale entropy analysis of pulse wave velocity for assessing atherosclerosis in the aged and diabetic. IEEE Trans. Biomed. Eng..

[20] Lin G.M., Haryadi B., Yang C.M., Chu S.C., Yang C.C., Wu H.T. (2017). Discrepancies between conventional multiscale entropy and modified short-time multiscale entropy of photoplethysmographic pulse signals in middle- and old-aged individuals with or without diabetes. Entropy.

[21] Wu H.T., Lee C.Y., Liu C.C., Liu A.B. (2013). Multiscale cross-approximate entropy analysis as a measurement of complexity between ECG R-R interval and PPG pulse amplitude series among the normal and diabetic subjects. Comput. Math. Methods Med..

[22] Wu H.T., Liu C.C., Lo M.T., Hsu P.C., Liu A.B., Chang K.Y., Tang C.J. (2013). Multiscale cross-approximate entropy analysis as a measure of complexity among the aged and diabetic. Comput. Math. Methods Med..

[23] Wu H.T., Yang C.C., Lin G.M., Haryadi B., Chu S.C., Yang C.M., Sun C.K. (2017). Multiscale cross-approximate entropy analysis of bilateral fingertips photoplethysmographic pulse amplitudes among middle-to-old aged individuals with or without type 2 diabetes. Entropy.

[24] American Diabetes Association (2014). Diagnosis and classification of diabetes mellitus. Diabetes Care.

[25] Lin S.L., Tung P.C., Huang N.E. (2009). Data analysis using a combination of independent component analysis and empirical mode decomposition. Phys. Rev. E Stat. Nonlinear Biol. Soft Matter Phys..

[26] Wu Z., Huang N.E., Long S.R., Peng C.K. (2007). On the trend, detrending, and variability of nonlinear and nonstationary time series. Proc. Natl. Acad. Sci. USA.

[27] Wu H.T., Lee C.H., Liu A.B., Chung W.S., Tang C.J., Sun C.K., Yip H.K. (2011). Arterial stiffness using radial arterial waveforms measured at the wrist as an indicator of diabetic control in the elderly. IEEE Trans. Biomed. Eng..

[28] Costa M., Goldberger A.L., Peng C.K. (2005). Multiscale entropy analysis of biological signals. Phys. Rev. E Stat. Nonlinear Biol. Soft Matter Phys..

[29] Li X., Yu S., Chen H., Lu C., Zhang K., Li F. (2015). Cardiovascular autonomic function analysis using approximate entropy from 24-h heart rate variability and its frequency components in patients with type 2 diabetes. J. Diabetes Investig..

[30] Pincus S., Singer B.H. (2014). Higher-order dangers and precisely constructed taxa in models of randomness. Proc. Natl. Acad. Sci. USA.

[31] Pan W.Y., Su M.C., Wu H.T., Su T.J., Lin M.C., Sun C.K. (2016). Multiscale entropic assessment of autonomic dysfunction in patients with obstructive sleep apnea and therapeutic impact of continuous positive airway pressure treatment. Sleep Med..

[32] Studinger P., Goldstein R., Taylor J.A. (2009). Age- and fitness-related alterations in vascular sympathetic control. J. Physiol..

[33] Corman B., Duriez M., Poitevin P., Heudes D., Bruneval P., Tedgui A., Levy B.I. (1998). Aminoguanidine prevents age-related arterial stiffening and cardiac hypertrophy. Proc. Natl. Acad. Sci. USA.

[34] McEniery C.M., O’Shaughnessy K.M., Harnett P., Arshad A., Wallace S., Maki-Petaja K., McDonnell B., Ashby M.J., Brown J., Cockcroft J.R. (2006). Variation in the human matrix metalloproteinase-9 gene is associated with arterial stiffness in healthy individuals. Arterioscler. Thromb. Vasc. Biol..

[35] O’Rourke M.F., Hashimoto J. (2007). Mechanical factors in arterial aging: A clinical perspective. J. Am. Coll. Cardiol..

[36] Jamwal S., Sharma S. (2018). Vascular endothelium dysfunction: A conservative target in metabolic disorders. Inflamm. Res..

[37] Loader J., Montero D., Lorenzen C., Watts R., Meziat C., Reboul C., Stewart S., Walther G. (2015). Acute Hyperglycemia Impairs Vascular Function in Healthy and Cardiometabolic Diseased Subjects: Systematic Review and Meta-Analysis. Arterioscler. Thromb. Vasc. Biol..

[38] Huang R., Abdelmoneim S.S., Nhola L.F., Basu R., Basu A., Mulvagh S.L. (2015). Relationship between glycosylated hemoglobin A1c and coronary flow reserve in patients with Type 2 diabetes mellitus. Expert Rev. Cardiovasc. Ther..

[39] Richman J.S., Moorman J.R. (2000). Physiological time-series analysis using approximate entropy and sample entropy. Am. J. Physiol. Heart Circ. Physiol..

